# Late-in-life surgery associated with Creutzfeldt-Jakob disease: a methodological outline for evidence-based guidance

**DOI:** 10.1186/1742-7622-10-5

**Published:** 2013-05-23

**Authors:** Mabel Cruz, Ignacio Mahillo-Fernandez, Alberto Rábano, Ake Siden, Miguel Calero, Henning Laursen, Kåre Mølbak, Javier Almazán, Jesus de Pedro-Cuesta

**Affiliations:** 1 Department of Clinical Neurosciences, Neurology Division, Karolinska Institutet, Stockholm, SE-141 86, Sweden; 2 Department of Applied Epidemiology, National Center for Epidemiology, Carlos III Institute of Health, C/ Sinesio Delgado 6, Madrid 28029, Spain; 3 Consortium for Biomedical Research in Neurodegenerative Diseases (Centro de Investigación Biomédica en Red sobre Enfermedades Neurodegenerativas - CIBERNED), Carlos III Institute of Health, C/ Sinesio Delgado 6, Madrid 28029, Spain; 4 Neuropathology Laboratory, Alzheimer’s Disease Center, Reina Sofia Foundation and Carlos III Institute of Health, Valderrebollo 5, Madrid, 28031, Spain; 5 Department of Spongiform Encephalopathies, National Microbiology Center, Ctra. Majadahonda-Pozuel1o, Km. 2,200, Majadahonda, 28220, Spain; 6 Neuropathology Laboratory, 6301. H:S Rigshospitalet, Blegdamsvej, 9, Copenhagen, DK-2100, Denmark; 7 Department of Epidemiology, Statens Serum Institut, Artillerivej, 5, Copenhagen, DK-2300, Denmark

**Keywords:** Etiology, Care, Creutzfeldt-Jakob disease, Epidemiology, Guidelines, Methods, Public health, Safety, Surgical procedures

## Abstract

**Background:**

There is increasing epidemiological evidence of etiological links between general surgery and sporadic Creutzfeldt-Jakob disease (sCJD) with long incubation periods. The purpose of this study was to identify specific surgical procedures potentially associated with sCJD to be targeted for preventive presurgical-intervention guidance.

**Results:**

We propose a three-step clinical guidance outline where surgical procedures associated with sCJD clinical onset – potentially more contaminant - are taken into account. Data on hospital discharges and surgical procedures were obtained from Danish and Swedish national in-patient hospital registries for 167 sCJD cases, onset 1987–2003, and for 835 matched and 2,224 unmatched population controls. Surgery was allocated to different life-time periods as previously reported, and frequencies were compared using logistic regression analysis. In the year preceding clinical onset, persons with sCJD underwent a statistically significant higher number of minor surgical interventions (OR (95% CI): 17.50 (3.64-84.24)), transluminal endoscopies (OR: 2.73 (1.01–7.37)) and gastrointestinal operations (OR: 3.51 (1.21–10.19)) compared to matched controls. Surgical discharges clustered towards clinical onset. These differences increased during the clinical period, with statistically significant higher frequencies for both endoscopies and minor surgery (OR: 13.91 (5.87-32.95), and for main surgical procedures (OR: 2.10 (1.00-4.39)), particularly gastrointestinal surgery (OR: 6.00 (1.83-19.66)), and surgery contacting skeletal muscle. Comparisons with unmatched controls yielded similar results for neurosurgery in the clinical period (OR: 19.40 (2.22-168.34)).

**Conclusions:**

These results suggest that some types of surgical procedures are associated with sCJD, after clinical onset or particularly just before onset. Selective planning of such surgery to minimize instrument/device contamination or quarantining might be feasible. Conditional to progress in sCJD etiological research, results are relevant for guidance development.

## Background

Creutzfeldt-Jakob disease (CJD) is a neurodegenerative disease associated with deposition in brain tissue of a pathological isoform (termed PrP^Sc^) of the normal cellular prion protein (PrP^C^). CJD exists in three forms, namely: sporadic (sCJD), of unknown etiology; acquired, whether variant (vCJD) or iatrogenic (iCJD); and caused by mutations in the gene encoding PrP [1]. Surgical transmission of CJD by tainted instruments has been experimentally demonstrated [2], and exposure to neurosurgical instruments recently used on a patient with CJD or incubating the disease constitutes a diagnostic criterion of iCJD in surveillance (http://www.cjd.ed.ac.uk/diagnosis.html). vCJD has been linked to both dietary exposure to bovine spongiform encephalopathy (BSE) and transfusion of blood from infected donors [3-5], but not to general or dental surgery [4,6]. The biological plausibility of vCJD transmission by surgery, and by neurosurgery in particular, is still being considered [7]. Insofar as sCJD is concerned, there is increasing, yet limited, epidemiological evidence of significant etiological links to: general surgery [8,9] with long incubation periods [9]; surgery of retina and peripheral nerves after shorter incubation intervals [10]; and recently - with conflicting results – to blood transfusion (BT) connected to surgery after a >10-year lag [11,12].

### State-of-the-art guidelines in EU member states

Recommendations for prevention of CJD transmission in medical settings may correspond either to public health guidelines (e.g., for blood donor deferral or instrument decontamination procedures), or to clinical guidelines (more oriented towards supporting decision-making by clinicians and surgeons). A recent report shows that a majority of EU Member States had official guidelines that included specific preventive measures covering situations where symptomatic and asymptomatic CJD patients or persons at potentially higher risk of CJD had to undergo surgery [13]. These included written advice for single-use equipment, destruction of contaminated equipment, decontamination of reusable instruments, use of protective clothing, storing and quarantining surgical instruments, as well as organizational recommendations. This report suggests that, should the new risk factors for sCJD be confirmed [8-11], further guidance will be needed [13].

### Rationale for guideline development

Within a background of guidance development for a potential prevention of CJD transmission by surgical instruments, this study would correspond to the first of six steps differentiated at a recent report on evidence-based public health advice for communicable diseases, i.e. the Preparatory, 0 Stage [14]. At such step, in addition to careful descriptive epidemiology of cases (CJD surveillance), data on incidents are collected. The present study focuses on potential incidents where instrument contamination may occur, using a case–control design. In consequence, we outline here a rationale to motivate the application of the case–control results at time of Developing Advice, Stage 3.

It must be stressed that considerable progress is yet needed in assessing evidence of etiological risk of sCJD from surgery or BT. An ad-hoc group, commissioned by the European Centre for Disease Prevention and Control (ECDC), cautiously concluded, after a formal review aimed to facilitate grading of scientific evidence in reports, that “the strongest evidence of positive associations for surgical procedures (SPs) and BT, were based on latency analysis at >10- or >20-year lag” and that “the positive findings in relation to SP and BT may indicate a true risk for sCJD, although there may be confounding of SPs by BT” [15]. The rationale proposed here should by no means be taken as a guide for advice before all formal steps of guidance development are followed. At such time, as suggested by the ECDC Chief Scientist “methods of classical epidemiology can be merged with the methodologies developed in evidence-based medicine to generate an evidence-based methodology for prevention and control of infectious disease” [14]. We believe that in the case of CJD such combined approaches – as shown in this study - can advantageously be early undertaken.

### Required epidemiologic data for SP assessment

Traditionally, assessment of CJD transmission risk levels for specific neurosurgical and ophthalmological procedures has been based on case reports and experimental data rather than on epidemiological associations. Proposed preventive measures in some EU Member States have addressed surgery to be undergone by patients with CJD early in the clinical period or being “at risk of sCJD or vCJD”, for SPs traditionally rated as high-risk, i.e., neurosurgical, ophthalmological and, in the specific case of vCJD, dental treatment, use of endodontic reamers, endoscopes in contact with nasal cavity, and maxillofacial surgery [13]. Attempts have been made to identify types of high-risk surgery that are more frequently performed at advanced incubation or after clinical onset of CJD, when tainted instrument infectivity can be higher. For instance, when compared to surgery on controls, statistically significant excess frequencies have been reported for coronary surgery in Denmark and Sweden, and for unspecified surgery at ≤6 years from onset in Germany [9,16]. In Japan, 4.5% of 760 sCJD patients underwent surgery before diagnosis and during the early clinical period; these procedures were neurological and ophthalmological in 0.8% and 1.8% of such patients, respectively [17]. It would seem that in the UK, ophthalmological surgery was performed at a higher than expected frequency during the early clinical period of CJD [18]. As seen, some attention has been given to the early clinical period in sCJD, but controlled studies are scarce.

### Study purpose

This study sought to outline principles which would be potentially useful for drawing up guidelines to prevent sCJD transmission, and describe surgical late-in-life experience of sCJD that would enable identification of the characteristics of SPs to which recommendations could be best applied. Further detailed description of the rationale lies outside the scope of this study.

## Results

### Outline of guidance

Algorithms used for planning any type of surgery having the potential to contaminate instruments incorporate the following two main variables: 1) patient-related infectious risk level (based on personal assessment, e.g., patients with *PRNP* mutations or family history of CJD, or cases where diagnosis of sCJD has been considered due to clinical symptoms), and, 2) the putative contaminating power of the planned procedure (extrapolated from the infectivity level assigned to tissues to be contacted by reusable instruments) [13,19]. In general, high-risk SPs encompass ophthalmological and neurosurgical procedures. Any potential change in SP assignment to SP risk-level categories would require theoretical and empirical motivation.

**Figure 1:**
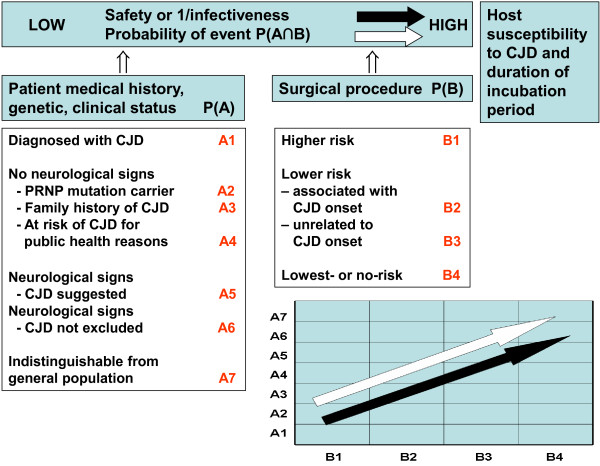
Basic components of a potential CJD transmission/induction process.

**Figure 2:**
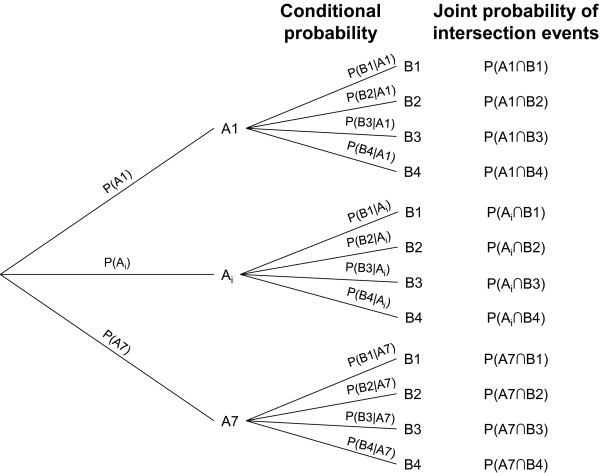
Probabilities of occurrence of different potential events associated with CJD transmission.

**Figure 3:**
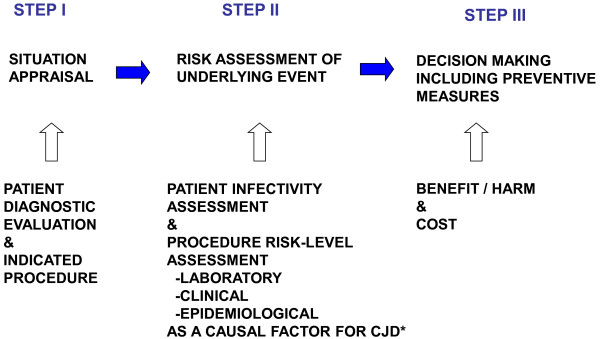
**Outline rationale behind an algorithm for estimating infectivity risk potentially associated with tissue remnants adhered to reusable instruments in a specific clinical situation, as defined by patient characteristics and planned surgery: Preparatory activity for theoretical development of the first two out of three steps.** I. Incident/event verification; II. Risk assessment; III. Developing advice/Implementation. Solid arrows indicate time; hollow arrows indicate information to be considered.

The rationale for prevention outlined in Figures 1, 2 and 3 and Table 1 combines the abovementioned patient- and SP-related two variables, by adding a patient-SP interaction factor. This factor seeks to identify a clinical situation, in which the intervention indicated corresponds to a category of SP more frequently conducted on CJD patients during the latter part of the incubation period or early clinical stage, at a time when the potential infectivity of reusable surgical instruments ought to be highest. There is considerable experimental evidence to support such high infectivity, e.g., inoculum infectivity increased with the progression of the infecting animal’s incubation period in experimental transmission of BSE and scrapie [20-23]. However, the association between specific SPs and CJD onset has not been clearly epidemiologically quantified epidemiologically. The effectiveness of any guidance in terms of accurately identifying and appropriately managing such patient-SP events would first depend on the use of suitable pattern-recognition criteria for high-risk events, defined in theory by: a) a combination of patient clinical characteristics; b) the indication of a SP classifed within a SP group for which an excess risk of CJD has been reported; for instance, surgery of *Digestive system and spleen*[9] or *Nose*/*throat* surgery [8]; and c) any SP identified from among those more frequently performed during late incubation or early clinical periods of CJD. Such ad-hoc SP tables and epidemiological criteria remain to be developed in future guidance work.

**Table 1:** Identification of basic information for algorithm building using three aetiological factors, potentially translated into criteria for action

**FACTOR 1. Patient term. Useful in STEPS I, II and III (Figure** 3**).**	**Probability**
-Patient has been clinically diagnosed with sCJD, vCJD, gTSE or iCJD	**PxP’**
-Patient has not been diagnosed with CJD* but is potentially infective due to:	**PxP’**
**a**) subclinical CJD* not being excluded;	
**b**) possibility of existence of prodromal symptoms but no CJD* diagnosis suspected;	
**c**) his/her being a symptom-free carrier of a pathologic mutation of *PRNP, *having a family history of CJD or being “at risk of CJD*” for public health purposes; or	
**d**) CJD* diagnosis suspected owing to clinical symptoms.	
where,	
**P**= daily incidence of indicated SP among patient’s age- and sex- group; and	
**P’ **= prevalence of infective persons among CJD diagnosed, **a**, **b**, **c **or **d (A1-7 **Figure 3).	
**FACTOR 2. SP putative risk level as a causal risk factor for a specific CJD form, i.e., vCJD, sCJD or iCJD, as defined by contacted tissue. Most useful in STEPS II and III, (Figure** 3**).**	
***-Higher***	**P**_**1**_**xP’**
***-Lower***	**P**_**2**_**xP’**
***-Lowest***	**P**_**3**_**xP’**
Categories defined under a new etiological body-system SP classification	
**P**_**n **_= daily incidence of the indicated SP among patient’s age- and sex- group; and,	
**P’ **= prevalence of infective persons among patient’s groups **(A1-7** Figure 3) undergoing the procedure.	
FACTOR 3. Patient/SP interaction term (SP with higher than expected incidence late in CJD course).** To be applied for situation appraisal, STEP I (Figure 3**).**	
**SPs to be identifed from associations reported by quality studies	
For SP categories defined under a body-system SP classification for use as a guide.	**(P**_**2**_**xP’)xA**_**2**_
	**(P**_**3**_**xP’)xA**_**3**_
**A**_**n **_parameter >1 determined by the magnitude of OR>1** for the indicated SP group or rubric, on termination of incubation period among population converting to CJD* versus population controls.	

* Here CJD constitutes a generic term for different forms of acquired Transmissible Spongiform Encephalopathy (TSE).

** Generated in our study from persons who in fact converted to clinical sCJD in Denmark and Sweden in 1987–2003. In future, this will probably be based on ORs reported in meta-analyses.

The impact of decision-making, using 3-factor algorithms based on the theory proposed in Figures 1, 2 and 3 and Table 1 should be estimated at a later stage of guidance development from empirical observations. This impact might be highest if applied to frequent SPs, i.e., *Lower*-risk SPs in STEP II, indicated for frequent clinical situations denoted in Figures 1–2 as A6 (patients with neurological signs, CJD not excluded); i.e. corresponding to P(A6∩B2) and P(A6∩B3) patient-SP events or alternatively to less frequent P(A3∩B2) surgical events involving patients with family history of CJD and SP associated with sCJD onset. The features of such SPs, namely, body-system group or rubric, obtained empirically from hospital statistics and records review and the associations defined by OR, could subsequently be integrated into recommendations for managing potentially contaminating events, particularly in cases where family antecedents or biological markers of CJD risk were lacking.

### Detection of non-random, joint events in which Ai may represent presence of CJD infectivity

Probabilities in Figure 2, when corresponding to independent A and B events, are as follows: *P*(*A* ∩ *B*) = *P*(*A*) · *P*(*B*) for joint probability (two events in conjunction) and *P*(*B*|*Α*) = *P*(*B*) for conditional probability, such that

$$P\left(B|Α\right)=\frac{P\left(A\cap B\right)}{P\left(A\right)}$$

Any situation in which *P*(*B*) proved to be higher when CJD was present P(*B*2|*A*1) than when it was absent, *P*(*B*2|*A*7), would be determined by presence of B2 SPs, namely, *P*(*B*2|*A*1) > *P*(*B*2|*A*7) or $\frac{P\left(B2|A1\right)}{P\left(B2|A1\right)}>1$.

Assuming that probabilities could correspond to incidences, joint events characterized by $\frac{P\left(B2|A1\right)}{P\left(B2|A7\right)}>1$ excess risk would be quantified by the odds ratio using logistic models.

As an example, we conduct such an exercise using a case–control material from Northern Europe.

### Study design

Since this was viewed as a cross-sectional observation study, a reported case–control design was adopted (see refs. [9,10] for details), including 167 probable or definite sCJD cases, 78 men and 89 women, residing in Denmark or Sweden and fulfilling established EuroCJD diagnostic criteria [24], with clinical onset during the period 1987–2003. Two sets of randomly chosen population controls were included, i.e., 835 matched control (MCs, 5:1 by gender, year, month of birth, and municipality of residence at death of the corresponding case), and 2,224 unmatched controls (UMCs). Mean and median ages at clinical onset or ID-2 were 67 and 68 years (range: 40–88) for cases and MCs, and 60 and 58 years (range: 40–99) for UMCs. For the purposes of delimiting lifetime intervals (late-in-life surgical history for cases and standard lifetime surgical history for controls), two time-windows (TWs) were defined using two operational dates, namely, date of death and date of clinical onset for cases, and the corresponding index dates, denoted index dates 1 and 2 (ID-1 and ID-2), for controls [9]. TW-4 covered the 1-year period preceding operational disease onset or ID-2. TW-5 encompassed the interval between date of operational disease onset and death in cases, and the corresponding period with the same mean duration, from ID-2 to ID-1, in controls. Earlier time-windows in life course, TW-1 to TW-3, not relevant here, were previously studied [9].

### Exposure ascertainment

For cases and controls, data on past hospital discharges [diagnoses, SP codes, and dates of admission and discharge] were obtained from the National Hospital In-patient Registers in Sweden and Denmark, and using discharge dates and personal identifiers, were then blindly allocated to subjects’ case or control status, one of the two TWs, and the two pooled TWs. SP codes were first categorized according to Swedish, Danish, and Nordic (NOMESCO-NCSP) SP classifications (for details see ref. [9]). Codes describing procedures that were not strictly surgical, e.g., delivery, and non-specific codes, e.g., “investigative procedures connected with surgery”, were omitted. The overall selected surgical experience of cases and controls captured by the present study corresponded to 598 repeated and unrepeated, and 335 distinct SP codes associated with 364 registered discharges during TWs 4 and 5.

The abovementioned 598 SPs were subsequently categorized into two major groups [9], namely, “main surgical procedures” and “subsidiary procedures”. The latter was a heterogeneous category, which included transluminal endoscopies, with or without biopsy, and minor invasive procedures (such as punctures, needle aspiration or biopsy, and superficial incisions) that, in general, were systematically included in the SP-classification chapters for “minor surgical procedures”. A few registered codes corresponding to blood transfusions in Denmark were dropped from the group of procedures studied. In order to obtain a picture of potential tissue remnants adhering to surgical instruments deemed to be reusable, “main surgical procedures” were reclassified by contacted tissue or structure using a reported method [25,26]. Endoscopies and minor surgical procedures were not reclassified.

An individual was deemed to be exposed to a specific type of surgery defined by body-system group or tissue/structure, during a specific window, when at least one hospital department discharge associated with at least one such surgical code under study was found at a date lying within the designated time interval, with surgical history in any other window being disregarded.

### Data-analysis

We quantified associations between subclinical or clinically manifested sCJD and specific categories of SP, using the odds ratio which, for infrequent exposures, approximately represents the ratio of the proportion of cases undergoing surgery to that of controls during an identical, mean, overall life-time interval. Statistical methods pertaining to the design of variables, latency intervals, multivariate models and procedures for calculation of 95% CIs replicated those used in earlier analyses [9,10]. Exposure, on the other hand, was taken as a binary variable only, i.e., exposed/unexposed.

### Ethics

Scientific evaluation and preliminary ethical clearance of the research proposal was done by the EU Research Commission’s ”Concerted Action QLRG3-CT-2002-81223”. The study was formally notified to the Danish Data Protection Agency (record no. 2003-41-3104) and approved by the Karolinska Institute Ethics Committee (South; report no. 452/02, 2002-12-02) and “Regionala Etiskprövningsnämnden” dnr 04-171T 2004-04-29 1).

Written consent was not given by patients for their information to be stored in the hospital data base and used for research since it was not needed. In Sweden, patients were never personally identified after register linkage took place by registries administrative officers. A data base was built after substituting at registries level each personal identification number by serial individual numbers. Data stored in official Swedish registries were used following legal regulations. The study conforms with Danish legislation requiring data management after notification to the Danish Data Protection Agency (Act on Processing of Personal Data - Act No. 429 of 31 May 2000). According to the Danish legislation, there is no need for scientific-ethical clearance of registry based studies, nor is there a need for written consent by cases (Act nr 593 of 14/06/2011, Section 10 paragraph 2. Additionally all CJD patients had died by the time of data collection. No biological or tissue data was studied.

### Case control study results

**Figure 4:**
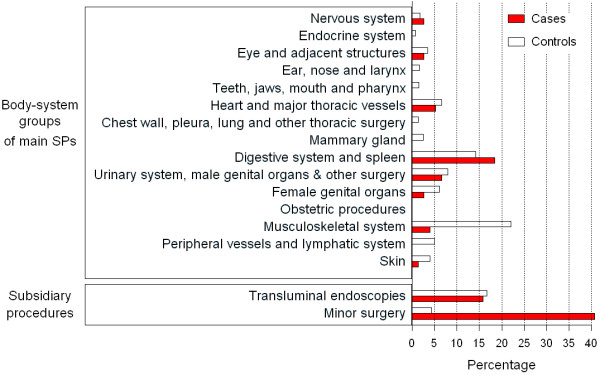
**Percentage distributions of selected SP codes associated with surgical discharges during TWs 4 and 5, classified by case–control status, body system, and type of subsidiary procedure (n=598).** SP categories yielding zero values are not shown.

The figures for repeated or unrepeated SPs associated with discharges during separate time-windows were as follows: 1) 36 for cases, 91 for MCs and 276 for UMCs, during TW-4; and, 2) 38 for cases, 56 for MCs and 101 for UMCs during TW-5. The distribution of the 598 SPs by body-system group and case–control status is depicted in Figure 4, which shows higher frequencies of minor and digestive system surgery among cases.

Table 2 shows the ORs for main and subsidiary SPs registered at discharge during TWs 4 and 5, both separately and pooled. Compared to MCs, cases in TW-4 registered statistically significant higher frequencies for subsidiary procedures (ORs (95% CI): 8.06 (3.15–20.63)), but not for main procedures targeting all body systems (OR: 1.71 (0.92–3.27)). Findings from comparisons with UMCs were similar. During TW-5, differences increased, with cases displaying statistically significant higher frequencies for endoscopies and minor surgery, (OR: 13.91 (5.87–32.95)), as well as for main procedures (OR: 2.10 (1.00–4.39)), than did MCs, with these figures being almost twice as high as those for UMCs. The overall pattern of late-in-life invasive or potentially invasive medical procedures in persons with diagnosed or undiagnosed sCJD in TWs 4 and 5, corresponds to an at least 2-fold frequency of main SPs and a 12-fold frequency of subsidiary examinations.

**Table 2:** Associations for main surgical and subsidiary procedures, for specific periods adjacent to clinical onset or ID-2, separately and combined

			**Unexposed**	Exposed*	**Surgery targeted by study**
Time window	Surgery under study	Subject	No. (%)	No. (%)	OR (95% CI)
Time window 4	Main surgical	*Case*	153 (91.6)	14 (8.4)	
	procedures	MC	793 (95.0)	42 (5.0)	1.71 (0.92–3.17)
		UMC	2,114 (95.1)	110 (4.9)	1.51 (0.80–2.64)
	Subsidiary	Case	155 (92.8)	12 (7.2)	
	procedures**	MC	826 (98.9)	9 (1.1)	8.06 (3.15–20.63)
		UMC	2,193 (98.6)	31 (1.4)	4.78 (2.27–9.49)
Time window 5	Main surgical	*Case*	156 (93.4)	11 (6.6)	
	procedures	MC	807 (96.6)	28 (3.4)	2.10 (1.00–4.39)
		UMC	2,175 (97.8)	49 (2.2)	3.14 (1.50–6.08)
	Subsidiary	*Case*	147 (88.0)	20 (12.0)	
	procedures	MC	827 (99.0)	8 (1.0)	13.91 (5.87–32.95)
		UMC	2,213 (99.5)	11 (0.5)	28.52 (13.09–65.24)
Time windows 4 and 5 combined	Main surgical	*Case*	143 (85.6)	24 (14.4)	
	procedures	MC	770 (92.2)	65 (7.8)	1.99 (1.21–3.30)
		UMC	2,071 (93.1)	153 (6.9)	2.05 (1.25–3.25)
	Subsidiary	*Case*	137 (82.0)	30 (18.0)	
	procedures	MC	819 (98.1)	16 (1.9)	11.77 (6.00–23.06)
		UMC	2,184 (98.2)	40 (1.8)	11.94 (6.94–20.45)

M. Matched control; UMC. Unmatched control.

ORs for comparisons with unmatched controls were adjusted for age, sex and country.

* At least one registered procedure.

** Endoscopies or minor surgery.

Patterns of surgical activity undergone by cases and controls are shown in Table 3 with a breakdown by body-system group. Compared to MCs, persons with sCJD in TW-4 underwent: a 20-fold higher frequency of minor surgical interventions (OR: 17.50 (3.64–84.24)); a statistically significant 3 to 4-fold higher frequency of endoscopic examinations and digestive-system surgery, with similar figures for comparisons with UMCs; and, in general, a trend-pattern of OR >1 for each SP group under study. The highest of the statistically non-significant differences was seen for surgery of heart and major vessels (OR: 5.75 (0.76–31.7)), when compared to UMCs. For TW-5, differences between cases and controls increased, with the highest differences being for minor surgery and neurosurgery (the latter based on two exposed cases) (OR: 165.1 (44.84–1,070)) and OR: 19.40 (2.03–15.94), respectively), and lower differences for gastrointestinal surgery (OR: 6.01 (2.03–15.94)), vis-à-vis UMCs. Based on lower numbers when compared to MCs, statistically significant, similar differences were seen for minor and gastrointestinal surgery.

**Table 3:** Number of cases and controls, and associations for surgery by body system group at discharge, for specific periods adjacent to onset or ID-2

			**Unexposed**	Exposed*	**Surgery targeted by study**
Time window	Body-system group	Subject	No. (%)	No. (%)	OR (95% CI)
Time window 4	Eye and adjacent	*Case*	166 (99.4)	1 (0.6)	
	structures	MC	833 (99.8)	2 (0.2)	2.50 (0.23–27.57)
		UMC	2,217 (99.7)	7 (0.3)	1.77 (0.09–10.51)
	Heart and major	Case	165 (98.8)	2 (1.2)	
	thoracic vessels	MC	830 (99.4)	5 (0.6)	2.00 (0.39–10.31)
		UMC	2,220 (99.8)	4 (0.2)	5.75 (0.76–31.47)
	Digestive system	*Case*	161 (96.4)	6 (3.6)	
	and spleen	MC	824 (98.7)	11 (1.3)	2.73 (1.01–7.37)
		UMC	2,191 (98.5)	33 (1.5)	2.22 (0.81–5.12)
	Urinary system,	*Case*	164 (98.2)	3 (1.8)	
	male genital organs	MC	831 (99.5)	4 (0.5)	3.75 (0.84–16.76)
	and other surgery	UMC	2,210 (99.4)	14 (0.6)	1.96 (0.44–6.31)
	Female genital	*Case*	87(97.8)	2 (2.2)	
	organs	MC	441 (99.1)	4 (0.9)	2.50 (0.46–13.65)
		UMC	1,136 (99.0)	12 (1.0)	1.92 (0.29–7.51)
	Transluminal	*Case*	161 (96.4)	6 (3.6)	
	endoscopies	MC	826 (98.9)	9 (1.1)	3.51 (1.21–10.19)
		UMC	2,199 (98.9)	25 (1.1)	2.57 (0.93–6.08)
	Minor surgical	*Case*	160 (95.8)	7 (4.2)	
	procedures	MC	833 (99.8)	2 (0.2)	17.50 (3.64–84.24)
		UMC	2,217 (99.7)	7 (0.3)	16.52 (5.34–51.15)
Time window 5	Nervous system	*Case*	165 (98.8)	2 (1.2)	
		MC	835 (100.0)	0 (0.0)	–
		UMC	2,222 (99.9)	2 (0.1)	19.40 (2.22–168.34)
	Digestive system	Case	161 (96.4)	6 (3.6)	
	and spleen	MC	830 (99.4)	5 (0.6)	6.00 (1.83–19.66)
		UMC	2,211 (99.4)	13 (0.6)	6.01 (2.03–15.94)
	Musculoskeletal	*Case*	165 (98.8)	2 (1.2)	
	system	MC	826 (98.9)	9 (1.1)	1.12 (0.23–5.33)
		UMC	2,205 (99.1)	19 (0.9)	1.21 (0.19–4.32)
	Skin	*Case*	166 (99.4)	1 (0.6)	
		MC	833 (99.8)	2 (0.2)	2.50 (0.23–27.57)
		UMC	2,223(100.0)	1 (0.0)	24.31 (0.95–622.5)
	Transluminal	*Case*	164 (98.2)	3 (1.8)	
	endoscopies	MC	828 (99.2)	7 (0.8)	2.14 (0.55–8.29)
		UMC	2,215 (99.6)	9 (0.4)	3.81 (0.82–13.32)
	Minor surgical	*Case*	149 (89.2)	18 (10.8)	
	procedures	MC	834 (99.9)	1 (0.1)	90.0 (12.01–674.2)
		UMC	2,222 (99.9)	2 (0.1)	165.1 (44.84–1,070)

M. Matched control; UMC. Unmatched control.

*ORs for comparisons with unmatched controls were adjusted for age, sex and country.

At least one registered procedure.

Only categories with exposed cases are shown.

Despite being small, absolute numbers and groups shown in the columns of Table 3 reveal surgical patterns for cases and controls. During TW-4, main SP exposures – i.e., endoscopies and minor surgery excluded - were similar, with two out of three of the most frequent invasive exposures corresponding to surgery on digestive, urinary or genital organs, 11/14 for cases and 19/26 for MCs. In TW-5, surgery undergone by cases appeared to differ, in that two out of three main SP exposures (6+2)/11 corresponded to surgery of digestive and nervous systems, and minor surgery, with 18 exposed persons, dominated the scene. Gastrointestinal surgery accounted for 12/25, i.e., approximately half of all main SP exposures undergone by cases during TWs 4 and 5.

The tissue/structure-related pattern of associations is set out in Table 4. No significant differences with MCs were observed in TW-4, except for “other tissues”. Higher frequencies of invasive procedures were seen in TW-5 for sCJD patients, with differences resulting from comparisons with UMCs proving statistically significant: OR 95% CI for SP contacting brain or dura mater (OR: 36.35 (1.86–711.57)) each; and, based on the same controls, skeletal muscle (OR: 2.76 (1.11–6.89)), and peritoneum (OR: 5.50 (2.07–14.66)).

**Table 4:** Associations for surgery by contacted tissue/structure, registered at discharge for specific periods adjacent to onset or ID-2

			**Unexposed**	Exposed*	**Surgery targeted by study**
Time window	Tissue/structure group	Subject	No. (%)	No. (%)	OR (95% CI)
Time window 4	Lymph nodes	*Case*	165 (98.8)	2 (1.2)	
		MC	831 (99.5)	4 (0.5)	2.50 (0.46–13.72)
		UMC	2,216 (99.6)	8 (0.4)	3.13 (0.72–13.69)
	Blood vessels	Case	165 (98.8)	2 (1.2)	
		MC	828 (99.2)	7 (0.8)	1.46 (0.33–6.48)
		UMC	2,217 (99.7)	7 (0.3)	3.88 (0.82–18.34)
	Anterior ophthalmic	*Case*	166 (99.4)	1 (0.6)	
	structures	MC	833 (99.8)	2 (0.6)	2.50 (0.23–27.77)
		UMC	2,217 (99.7)	7 (0.3)	1.77 (0.24–13.29)
	Skeletal muscle	*Case*	164 (98.2)	3 (1.8)	
		MC	813 (97.4)	22 (2.6)	0.68 (0.20–2.29)
		UMC	2,169 (97.5)	55 (2.5)	0.57 (0.17–1.89)
	Peritoneum	*Case*	162 (97.0)	5 (3.0)	
		MC	826 (98.9)	9 (1.1)	2.78 (0.93–8.32)
		UMC	2,194 (98.7)	30 (1.3)	2.13 (0.80–5.66)
	Other tissues	*Case*	161 (96.4)	6 (3.6)	
		MC	826 (98.9)	9 (1.1)	3.51 (1.24–9.96)
		UMC	2,190 (98.5)	34 (1.5)	2.02 (0.83–4.93)
Time window 5	Brain	*Case*	165 (98.8)	2 (1.2)	
		MC	835 (100.0)	0 (0.0)	
		UMC	2,223 (100.0)	1 (0.0)	36.35 (1.86–711.57)
	Dura mater	*Case*	165 (98.8)	2 (1.2)	
		MC	835 (100.0)	0 (0.0)	
		UMC	2,223 (100.0)	1 (0.0)	36.35 (1.86–711.57)
	Lymph nodes	*Case*	166 (99.4)	1 (0.6)	
		MC	831 (99.5)	4 (0.5)	1.25 (0.14–11.26)
		UMC	2,218 (99.7)	6 (0.3)	2.07 (0.28–15.05)
	Skeletal muscle	*Case*	161 (96.4)	6 (3.6)	
		MC	825 (98.8)	10 (1.2)	3.13 (1.13–8.65)
		UMC	2,196 (98.7)	28 (1.3)	2.76 (1.11–6.89)
	Peritoneum	*Case*	161 (96.4)	6 (3.6)	
		MC	832 (99.6)	3 (0.4)	10.00 (2.49–40.15)
		UMC	2,210 (99.4)	14 (0.6)	5.50 (2.07–14.66)
	Other tissues	*Case*	166 (99.4)	1 (0.6)	
		MC	826 (98.9)	9 (1.1)	0.56 (0.07–4.41)
		UMC	2,214 (99.6)	10 (0.4)	1.66 (0.19–14.86)

M. Matched control; UMC. Unmatched control.

ORs for comparisons with unmatched controls were adjusted for age, sex and country.

* At least one registered procedure.

Only categories with exposed cases are shown.

Table 5 lists the types of intervention undergone by sCJD patients for which significant excess risk was observed in TW-4 or TW-5. Insofar as main SPs were concerned, cases in TW-4 registered a high number and a considerable range of gastrointestinal interventions, as well as gynecological and urological procedures, with coronary surgery being undergone by the two cases for whom surgery of heart and major vessels had been recorded. During the clinical period, in contrast, main SPs displayed a distinct, more uniform pattern, with percutaneous gastrostomy proving the most frequent intervention and neurosurgical interventions, craniotomy/biopsy and ventriculostomy being recorded in different patients. SP patterns in TWs 4 and 5 also differed by contacted tissue/structure, with “other tissues” in TW-4 and “skeletal muscle”, “peritoneum”, “brain” and “dura mater” in TW-5 being the most representative features. Patterns were rather similar for subsidiary procedures, such as transluminal endoscopies exploring the digestive and urinary systems, and in the case of Sweden alone, mainly lumbar punctures.

**Table 5:** Late preclinical and clinical patterns of surgical history for sCJD cases

**Time window and body-system or tissue group**	**Number of SP codes**	**Codes**	**Rubric**	Stay duration in days (*mean)
Time window 4				
Digestive system and spleen	1	JGA0	Endoscopic polypectomy in rectum	1
	1	JDB10	Percutaneous gastrostomy	12
	1	JAB30	Repair of inguinal hernia using prosthetic material	4
	2	5353, JKA21	Laparoscopic cholecystectomy	2*
	1	JGB10	Partial proctectomy and end colostomy	86
	1	JHA00	Anal or perianal incision	90
Heart and major thoracic vessels	1	FMD10	Replacement of aortic valve using biological prosthesis	8
	1	FNC10	Aorto-coronary venous bypass with single distal anastomosis	8
	1	FXB00	Intraoperative partial cardiopulmonary bypass	8
	1	FNG00	Dilatation of coronary artery	1
Transluminal endoscopies	4	UJD02	Gastroscopy	16
	2	UJF32	Coloscopy	23
	2	UJF42	Flexible sigmoidoscopy	1
	2	9006	Cystoscopy	10
Minor surgery	6	TAB00	Lumbar puncture	15
	1	TJJ00	Percutaneous needle biopsy of liver	34
	1	TPH20	Insertion of central venous catheter through subclavian or brachiocephalic vein	1
Other tissue	1	KJGA05	Endoscopic polypectomy in rectum	1
	1	KKED22	Transurethral resection of prostate	7
	1	KLCA13	Curettage of cervix and body of uterus	2
	1	KKCD02	Transurethral resection of bladder	4
	1	JHA00	Anal or perianal incision	90
	1	7280	Curettage of uterus for biopsy or as therapy	2
Time window 5				
Nervous system	1	AAA10	Biopsy through craniotomy	2
	1	AAF00	Ventriculostomy	2
Digestive system and spleen	4	JDB10	Percutaneous gastrostomy	17*
	1	JDB00	gastrostomy	30
	1	4644	Excision of sigmoid colon	10
Transluminal endoscopies	2	UJD02	Gastroscopy	18
	1	4042	Laparoscopy	10
	1	UKC02	Cystoscopy	2
Minor surgery	21	330, TAB00	Lumbar puncture	38
	1	TPW10	Implantation of vascular injection port	37
Brain	1	KAAA10	Biopsy via craniotomy	2
	1	AAF00	Ventriculostomy	2
Dura mater	1	KAAA10	Biopsy via craniotomy	2
	1	AAF00	Ventriculostomy	2
Peritoneum	4	JDB10	Percutaneous gastrostomy	17
	1	JDB00	Gastrostomy	30
	1	4644	Excision of sigmoid colon	10
	1	4042	Laparoscopy	10

Rubrics of surgical procedures significantly more frequently conducted in cases than in controls during the year preceding clinical onset* and during the period from onset to death, by body-system or tissue/structure group.

*For TW-4, heart and major vessel surgical procedures in cases are also listed.

Mean duration was calculated regardless of whether or not procedures were performed during the same hospital stay.

Certain surgery not significantly associated with sCJD but present in discharges post-cystoscopy, e.g., *percutaneous removal of calculus from kidney or urether*, was not listed here.

**Figure 5:**
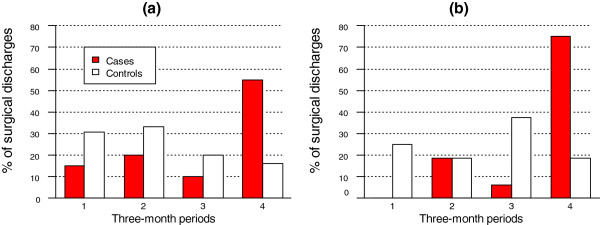
**Temporal distribution of hospital discharges associated with surgical interventions during TW-4, i.e., during the year preceding clinical onset in cases or ID-2 in matched controls: (a) discharges after main surgical procedures (n=20 for cases and n=75 for MCS); and (b) for discharges associated with endoscopies or minor surgical procedures (n=16 for cases and n=16 for MCs).** Quarters indicated in calendar time order.

Figure 5 depicts the quarterly distribution of surgical discharges for main and subsidiary procedures undergone by cases and MCs in TW-4. Twenty discharges of cases and 75 discharges of MCs were associated with main SPs, with heterogeneity tests p=0.033 and p=0.097, respectively. Sixteen discharges associated with subsidiary procedures were observed for each group, with heterogeneity significance (χ^2^ test) being p=0.000 for cases and p=0.788 for MCs. Discharges connected with main and subsidiary procedures clustered towards onset in cases (n=20 for main SP, and n=16 for subsidiary procedures) but not towards ID-2 in MCs (n=75 for main SP and n=16 for subsidiary procedures) . No clear patterns were seen for distribution by quartiles in TW-5 (data not shown).

## Discussion

Clinical guidance together with increasing use of disposable surgical instruments, control of inadvertent migration of potentially tainted instruments between surgical sets, and improvement of the processing and decontamination cycle, are considered to be the most important preventive measures to minimize potential CJD transmission by invasive medical procedures. This study shows that patients with sCJD in the clinical stage, TW-5, undergo a considerably high frequency of main SPs, for the most part neurosurgical and gastrointestinal, and minor SPs in particular, mostly in the form of lumbar punctures. It also suggests that, prior to clinical onset, TW-4, subsidiary procedures such as lumbar punctures and endoscopies, particularly of the digestive tract, as well as gastrointestinal surgery and a miscellaneous group of main SPs, occur at a higher than expected frequency for individuals who will develop sCJD. Identification of such potentially higher-risk events, particularly prior to CJD diagnosis, might well constitute a priority in clinical settings. Evidence-based guidance development will require consideration of multiple aspects of the potential transmission process and impact of preventive measures.

While the present study is unique in its use of registry-based assessment of surgical histories, randomly chosen controls and accurate life-time measurement, its limitations include a lack of access to hospital surgical records -offset in part by the high validity of registered SPs- [27] and exclusion of out-patient surgery and invasive diagnostic procedures. Lack of data on surgical judgement and neurological diagnostic processes hampers assessment of surgical indication and hospital preventive measures taken. However, case-selection and case–control differential SP registration are unlikely [9]. However, the younger age of controls might perhaps bias comparisons of surgery of fertile age when using UMCs.

Lumbar punctures, frequently performed in out patient care, out and gastrostomy form part of the routine diagnosis and care of demented patients in both countries. Their presence in TW-4 casts serious doubts on the accuracy of the operational time point of clinical onset of sCJD, indicating that both the onset of clinical manifestations and the neurological or medical examinations, frequently conducted at different hospitals, predated the operational date of clinical onset adopted after examining only one or two selected hospital records. As a consequence, our results may have overestimated differences between cases and controls in TW-4 and underestimated those in TW-5, particularly as regards endoscopies, lumbar punctures and digestive system surgery, which constitute a shared pattern. In practice, this would mean that surgery undergone prior to clinical onset would be reduced to almost expected levels vis-à-vis rates in controls, and that the excess medical procedures and identification of high-risk predictors would be ascribed to the early clinical period. Laske *et al*. reported statistically non-significant ORs of 6.97 and 3.39 for surgery up to 0.5 and 1 years prior to clinical onset [16].

Only one patient in TW-4 and none in TW-5 underwent ophthalmological surgery, thereby differing from the higher frequencies suggested by British and Japanese studies [17,18]. It is difficult to establish whether this negative finding is due to our study’s comparatively lower statistical power or to high clinical standards in Denmark and Sweden aimed at avoiding unnecessary interventions in cases where both cataract and cortical visual symptoms are possibly present at clinical onset of CJD. The excess of gastrointestinal surgery in TW-5 can be presumed to be mainly related to care, which, in TW-4, might in part be attributable to sCJD-related complaints that may either have unveiled other ailments or, alternatively, misled clinicians facing a patient who might not have been cognitively intact before sCJD diagnosis. Interestingly, Laske *et al.* deemed surgery and other stressful events to be a trigger of sCJD clinical onset within six months [16].

The statistically non-significant higher frequency of coronary surgery in TW-4 is a particularly interesting finding, since the only statistical significant association for specific body systems observed during the nine years preceding onset/ID-2 with OR 2.03 and 2.58 as compared to MCs and UMCs, respectively; and at >1 year before onset/ID-2, was surgery of heart and major vessels, i.e., coronary surgery [9]. The recalculated figures for TWs 3 and 4, i.e., the 10-year period, using a binary variable for exposure, were OR 2.27 (1.03–5.02) and 3.30 (1.42–7.00), when compared to MCs and UMCs, respectively. Our results suggest that coronary surgery is more frequently conducted within a several-year period predating sCJD onset, adding further fuel to the debate on reuse of angioplasty catheters [28,29].

In Denmark and Sweden, digestive tract and peripheral vessel surgery and interventions pertaining to peritoneum, skeletal muscle and “other tissues” constituted risk factors for sCJD, when conducted at considerable lags [9]. Furthermore, the results show that most are also associated with sCJD after clinical onset. Gastrointestinal and orthopedic SPs undertaken at end of life in persons with sCJD might generate infective remnants of skeletal muscle, “other tissues” and abdominal structures adhering to instruments, consistent both with the “*lower*” infectivity level assigned to such tissue by the WHO tissue infectivity tables [26] and with results of the abovementioned experimental observations [20-23]. Accordingly, warnings proposed for neurosurgical procedures in general, and for ophthalmological SPs in Japan and the UK in particular [17,18], may also apply to procedures overrepresented late in life among individuals with sCJD. To recapitulate, experimental, clinical and epidemiological observations would support the notion that algorithms designed to assess risk when planning surgical interventions in some EU Member States should include neurosurgical, ophthalmological and gastrointestinal procedures among those with higher infective potential.

The results of this and two earlier studies [9,10] cover all in-patient registered surgery undergone by persons with sCJD in Denmark and Sweden, and enable an overall picture to be formed of the relationship between surgery and sCJD since the early 1970s, which could well be valid for populations across the industrial world. Surgery is, in general, only directly associated with the disorder, and displays the following three, principal, etiologically different patterns: 1) surgery potentially causing sCJD, mainly conducted 20 or more years before disease onset; 2) surgery potentially or definitely caused by sCJD, mainly conducted at some point, early or otherwise, during the clinical course of the disease; and, 3) coronary surgery conducted in the 10 years preceding onset, which we contend represents a confounding effect of vascular risk factors acting concurrently as a cause of both coronary artheriosclerosis and sCJD. Subject to confirmation being obtained from independent studies, all three types of associations raise points relevant for designing specific guidelines for the prevention of sCJD transmission in medical settings.

In surgical practice, national and local traditions vary. Our results may not necessarily mimic the findings of prior studies nor be replicated in studies pertaining to other countries or time intervals. Clinical guidance for prevention of CJD transmission in medical settings calls for the study of recent surgical practice in countries where application of the relevant recommendations is envisaged, as well as analysis of temporarily overlooked yet relevant patient-SP specific events, denoted as incidents *a posteriori*. Despite a non-neurosurgical incident implicating consecutive use of same instrument by two persons diagnosed with sCJD has never been reported, incident assessment and management constitutes a distinct, mixed (clinical and public health) element of guidance for CJD prevention as current guidelines frequently show [13].

## Conclusions

In persons with sCJD certain types of end-of-life surgical interventions and medical examinations tend to be particularly frequent, e.g., digestive tract endoscopies, abdominal surgery and, possibly, surgery of nervous system, bone and coronary vessels. Their nature and indication should be further investigated using clinical records, with the aim of avoiding unnecessary procedures and planning interventions in accordance with evidence-based rules for the prevention of a potential CJD induction in medical settings.

## Competing interests

The authors declare that they have no competing interests.

## Authors’ contributions

IMF and JdPC conceived the study. M Cruz and KRM collected the data and validated diagnoses. IMF designed the statistical and probabilistic methods and conducted the statistical analysis. AR reclassified surgical codes. JdPC has full access to all of the data in the study and takes responsibility for the integrity of the data and the accuracy of the data analysis. M Cruz and JdPC produced the first manuscript. All authors contributed to and approved the manuscript final version.

## EUROSURGYCJD group members

Spanish team: Javier ALMAZÁN, María J. BLEDA, Miguel CALERO, Ignacio MAHILLO, Pablo MARTÍNEZ-MARTÍN, Jesús de PEDRO-CUESTA (coordinator), Alberto RÁBANO. Danish team: Gerhard FALKENHÖRST, Henning LAURSEN, Kåre MØLBACK. Finnish team: Jussi KOVANEN. Swedish team: Mabel CRUZ, Åke SIDEN.
